# *Dbx1* Is a Direct Target of SOX3 in the Spinal Cord

**DOI:** 10.1371/journal.pone.0095356

**Published:** 2014-04-21

**Authors:** Nicholas Rogers, Dale McAninch, Paul Thomas

**Affiliations:** Discipline of Biochemistry, School of Molecular and Biomedical Sciences, University of Adelaide, Adelaide, Australia; Universitat Pompeu Fabra, Spain

## Abstract

SoxB1 sub-family of transcriptional regulators are expressed in progenitor (NP) cells throughout the neuroaxis and are generally downregulated during neuronal differentiation. Gain- and loss-of-function studies indicate that *Sox1*, *Sox2* and *Sox3* are key regulators of NP differentiation and that their roles in CNS development are largely redundant. Nevertheless, mutation of each SoxB1 individually results in a different array of CNS defects, raising the possibility that SoxB1 proteins have subtly different functions in NP cells. To explore the mechanism of SOXB1 functional redundancy, and to identify genes that are most sensitive to loss of the *Sox3* gene, we performed genome wide expression profiling of *Sox3* null NP cells. Nineteen genes with abnormal expression were identified, including the homeobox gene *Dbx1*. Analysis of *Sox3* null embryos revealed that *Dbx1* was significantly reduced in the neural tube and developing brain and that SOX3 bound directly to conserved elements associated with this gene in cultured NP cells and *in vivo*. These data define *Dbx1* as a direct SOX3 target gene whose expression, intriguingly, is not fully rescued by other SOXB1 transcription factors, suggesting that there are inherent differences in SOXB1 protein activity.

## Introduction

SOX3 is a member of the SOX (Sry-related HMG box) family of transcription factors, of which 20 members have been identified in mammals. *SOX* genes generally have developmentally-regulated expression and play important roles in cell specification, self-renewal and differentiation in a broad range of embryonic contexts [1]. Within the developing central nervous system (CNS), *Sox3*, and the closely related genes *Sox1* and *Sox2* (which together make up the SoxB1 subgroup), are expressed in neuroprogenitor cells throughout the neuroaxis and are down regulated upon differentiation [2], [3]. Overexpression studies in chick embryonic spinal cords and cultured murine neuroprogenitor (NP) cells indicate that SOXB1 proteins function as inhibitors of neurodifferentiation and that they have overlapping roles in this process [2], [4]. SOXB1 group functional redundancy is also supported by loss-of-function studies in mammals. *Sox3* null mice exhibit specific CNS defects within the hippocampus, corpus callosum and hypothalamus despite the widespread *Sox3* expression in NP cells throughout the developing brain [5], [6]. In addition, humans with polyalanine tract expansion mutations in *SOX3* have a relatively mild phenotype that includes infundibular hypoplasia, hypothamalic-pituitary axis dysfunction and incompletely penetrance of intellectual disability [7], [8]. CNS-specific deletion of *Sox2* or *Sox1* in mice also results in regionally-restricted defects as opposed to a general NP phenotype [9], [10], [11], [12], [13].

While these studies provide strong support for SOXB1 functional redundancy, the existence of congenital CNS defects in *Sox3*, *Sox2* and *Sox1* single gene mutant mice also indicates that there is a specific requirement for each SOXB1 factor in a (relatively small) subpopulation of NP cells. This phenomenon can be explained by at least three possibilities. Firstly, given that subtle differences in *Sox1*, *Sox2* and *Sox3* gene expression have been identified in the developing CNS [3], [14], [15], it is possible that some NP cells express only one SOXB1 gene. Deletion of the single expressing SoxB1 gene in these cells would likely cause a specific developmental defect due to the complete absence of SOXB1 activity. However, while this is an attractive hypothesis, NP cells expressing only one SOXB1 gene have not been definitively identified. Furthermore, contrary to this possibility, structures that express more than one SOXB1 gene can be defective in single gene mutants. For example, development of the infundibulum is abnormal in *Sox3* null embryos despite expression of *Sox2* in this structure [5]. A second possibility is that SOXB1 proteins can bind to and regulate the same set of target genes but that each factor has a unique preference for specific targets over others due to their inherent sequence-specific DNA binding activity. In this scenario, deletion of a single SoxB1 gene would only affect the expression of SOXB1 targets for which it has a uniquely high affinity. Consistent with this idea, recent ChIP-seq analysis has revealed extensive overlap between SOX2 and SOX3 binding sites in NP cells [4]. A third possibility is that the SOXB1 proteins are functionally identical and that changes in the dosage of SOXB1 protein, as a whole, in an NP cell will alter the expression of some target genes. A prediction of this hypothesis is that mutation of individual SoxB1 genes would result in near identical or at least similar phenotypes. However, on the contrary, the phenotypes of single gene mutations are quite distinct and have few overlapping features.

To explore the mechanism of SOXB1 functional redundancy, and to identify genes that are most sensitive to loss of the *Sox3* gene, we performed genome wide expression profiling of *Sox3* null NP cells. Nineteen genes with abnormal/delayed expression were identified that included the homeobox gene *Dbx1*. *In vivo* analysis of *Sox3* null embryos revealed that *Dbx1* was significantly reduced in the neural tube and developing brain and that SOX3 bound directly to conserved elements associated with this gene in cultured NP cells and *in vivo*. These data define *Dbx1* as a direct SOX3 target gene whose expression, intriguingly, is not fully rescued by other SOXB1 transcription factors, suggesting that there are inherent differences in SOXB1 protein activity.

## Methods

### ES Cell Generation

*Sox3* null embryonic stem cells were previously targeted as described in (Hughes et al 2013). *Sox3* null mice have been published previously [5].

### ES Cell Culture and Neurodifferentiation

R1 ES cells were maintained on irradiated MEFs in standard conditions and neurodifferentiated as monolayers using N2B27 as previously described [16].

### Microarray and qRT-PCR

RNA from differentiated cells was extracted using a RNeasy mini kit (Qiagen) and cDNA generated using a High Capacity RNA-to-cDNA kit (ABI). Expression profiling was performed on four wild type and four *Sox3* null Day 4 samples using Affymetrix GeneChip Mouse Gene 1.0 ST Arrays (full dataset available at Gene Expression Omnibus: GSE53760). 2 way-ANOVA, using batch as a factor, was used to identify the significantly regulated genes. qRT-PCR validation was performed previously described [17].

### Immunohistochemistry

IHC was performed as described previously [3].

### Chromatin Immunoprecipitation (Chip)

In vitro ChIP was performed as per [4] using 5 ug of SOX3 or IgG antibodies, with Dynabeads blocked over night with 200 ug BSA, 200 ug yeast tRNA and 200 ug glycogen. In vivo ChIP was performed with wild type 10.5 dpc embryonic heads, disassociated with scalpel blades and fixed for 10 minutes using the above protocol. ChIP samples were analysed by qPCR (StepOne Plus, Applied Biosystems) using Fast SYBR (Life Technologies). Signals were considered positive when 2∧-[Ct_sample_ (non-enriched region) - Ct_sample_ (peak region)] was >2 following normalisation to Ct_IgG_ and Ct_Input_ of the corresponding qPCR run.

### Ethics Statement

Animal experiments were approved by the University of Adelaide Animal Ethics Committee (project number: S-2012-242). All studies were conducted in accordance with the principles of animal replacement and reduction and experimental refinement.

## Results

### Sox3 Expression during In vitro Neural Progenitor Differentiation

**Figure 1:**
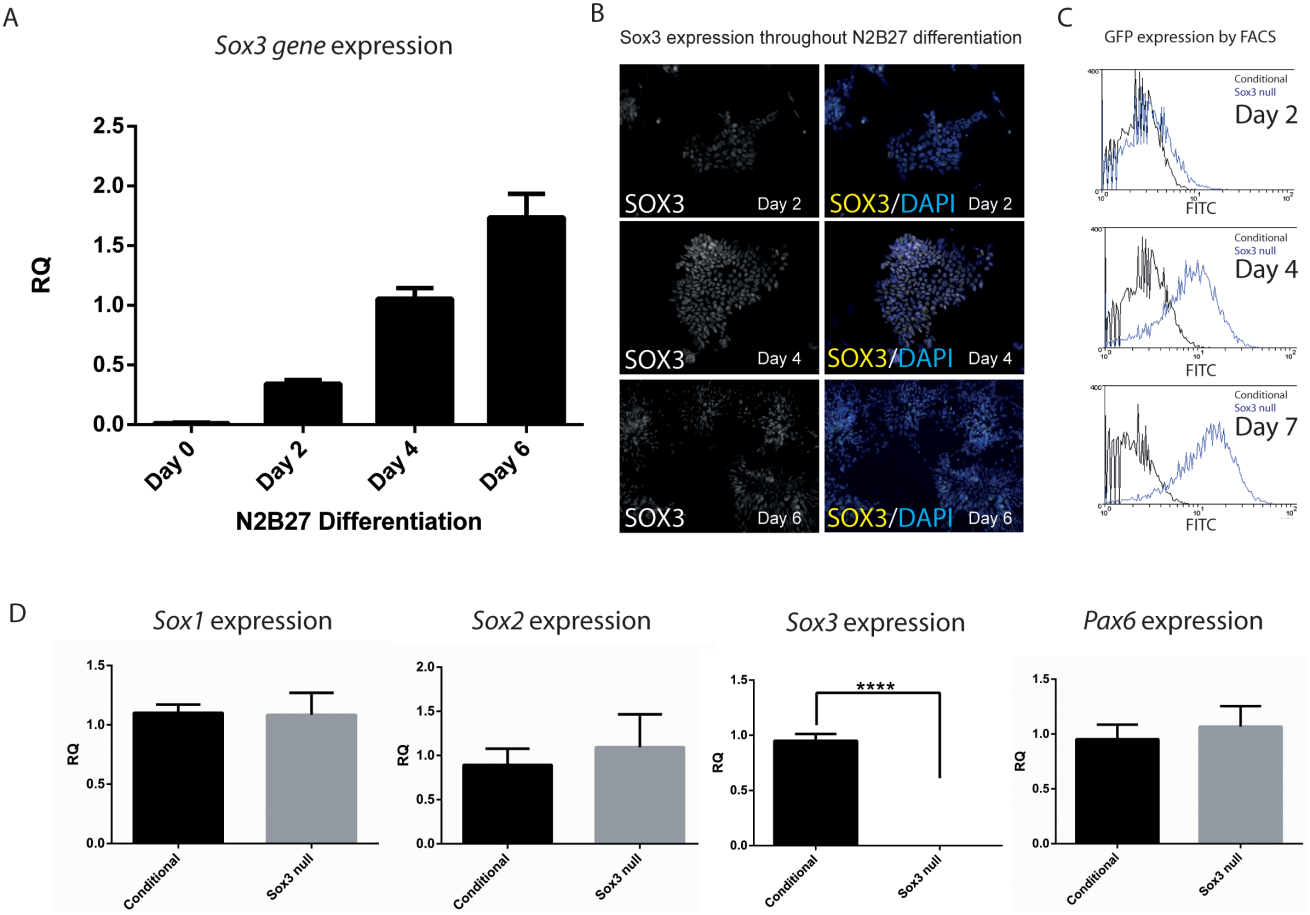
Sox3 expression in neural progenitor differentiation. A: Time course of *Sox3* gene expression levels by qRT-PCR throughout N2B27 differentiation (normalised to day 4 *Sox3* expression, n = 3). B: IHC of SOX3 showing expression at days 2, 4 and 6 of neuronal differentiation. C: GFP expressing cells sorted by FACS within *Sox3* null N2B27 differentiated ES cells at days 2, 4 and 7. D: qRT-PCR of *Sox1, Sox2, Sox2 and Pax6* expression at day 4 of N2B27 differentiation (n≥4). RQ: relative quantification normalised to β-actin.

To begin to identify SOX3 target genes in NP cells, we utilised the N2B27 culture system, which has previously been shown to generate NP cells from ESC with high efficiency [16]. To characterise *Sox3* expression during N2B27 neuroinduction, we performed qRT-PCR (Fig. 1A) and immunohistochemistry analysis (Fig. 1B;[3]). At the mRNA level, *Sox3* expression was virtually undetectable in ESC (Day 0). *Sox3* expression was upregulated by Day 2, and increased further at Day 4 and Day 6. Consistent with these data, weak expression of SOX3 protein was evident in Day 2 cultures. By Day 4, robust expression of SOX3 was detected in most cells. At Day 6, robust expression of SOX3 could still be detected in most cells, although some cells (particularly those at the periphery of colonies) had begun to downregulate SOX3.

To investigate the impact of *Sox3* deletion on NP differentiation, we performed N2B27 neuroinduction using our previously-generated *Sox3* null ES cells that contain a GFP reporter knock-in allele [17]. FACS analysis of Day 4 NP cultures revealed that the majority (over 60%) of cells were GFP+, which is comparable to the proportion of cells that are SOX3+ in the WT NP cultures (Figure 1B,C). To determine whether the loss of *Sox3* has a major impact on induction of neural progenitors, expression of neural progenitor markers (including other *SoxB1* genes) was analysed by qRT-PCR. No significant difference in *Sox1, Sox2* or *Pax6* expression was detected in samples utilized for microarray analysis, at Day 4 (Fig. 1D). No change in expression of the other SoxB1 subgroup members was also observed on Day 6 (data not shown). As expected, *Sox3* expression was not detected in the mutant samples (Fig. 1E). Together these data indicate that *Sox3* is rapidly upregulated during N2B27 neurodifferentiation and that loss of *Sox3* does not overtly affect NP induction across this timeframe, probably due to functional redundancy with other SOXB1 proteins.

### Identification of SOX3 Target Genes

**Figure 2:**
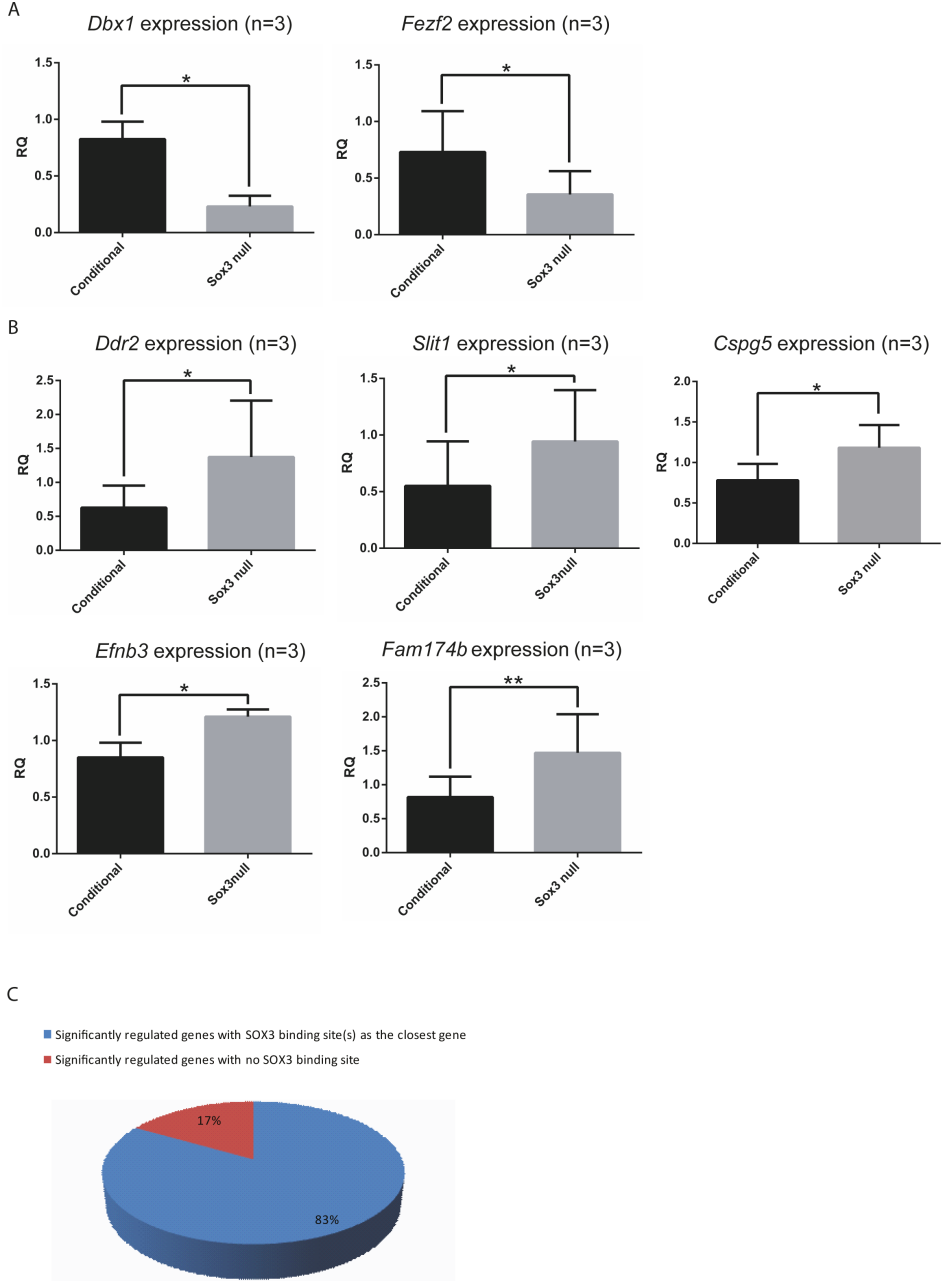
Validation of putative Sox3 targets. A: qRT-PCR validation of gene expression levels of down regulated genes, *Dbx1* and *Fezf2* (n = 3). B: qRT-PCR validation of gene expression levels of up regulated genes *Ddr2*, *Slit1*, *Cspg5, Efnb3* and *Fam174b* (n = 3). C: Percentage of significantly regulated genes with SOX3 binding sites, as closest gene. RQ: relative quantification normalised to β-actin.

To identify putative SOX3 target genes, we compared the global gene expression profile of Day 4 NP cell cultures derived from *Sox3* null cells and a control cell line containing a conditional *Sox3* allele [17]. Microarray analysis was performed in quadruplicate using 4 biological replicates from two independent experiments. A total of 19 genes had significantly (p≤0.05) altered expression with a fold change of 1.4 or more, as determined by batch corrected two-way ANOVA analysis (Table 1). Fifteen and 4 genes were upregulated and downregulated, respectively. *Sox3* provided an internal control for this experiment and, as expected, this gene was significantly downregulated in the *Sox3* null samples. Further validation of a subset of the misregulated genes was conducted using qRT-PCR on independent biological samples. *Dbx1 (p = 0.049)* and *Fezf2 (p = 0.0228)* showed significant down regulation in mutant cells (Fig. 2A). Significantly elevated expression of *Efnb3 (p = 0.0229), Cspg5 (p = 0.0251), Fam174b (p = 0.0065), Ddr2 (p = 0.0386)* and *Slit1 (p = 0.0456)* was also confirmed in the independent samples. These data identifies a number of potential SOX3 targets in cultured NP cells.

**Table 1:** Table of significantly regulated genes with a fold change equal to or greater than 1.4 in Day 4 N2B27 differentiated ES cells.

Gene Symbol	RefSeq	stepup p-value	Fold-Change	Fold-Change (Description)	Independently Validated
Sox3	NM_009237	0.01	−4.10	KO down vs WT	Yes
Cspg5	NM_001166273	0.02	1.49	KO up vs WT	Yes
Ddr2	NM_022563	0.02	1.83	KO up vs WT	Yes
Slit1	NM_015748	0.04	1.87	KO up vs WT	Yes
Tagln3	NM_019754	0.04	1.54	KO up vs WT	
1200009O22Rik	NM_025817	0.04	1.41	KO up vs WT	
Tmem163	NM_028135	0.04	−1.45	KO down vs WT	No
Slc44a5	NM_001081263	0.05	1.44	KO up vs WT	No
Fezf2	NM_080433	0.05	−1.61	KO down vs WT	Yes
Fam174b	NM_001162532	0.05	1.64	KO up vs WT	Yes
Fgfr3	NM_008010	0.05	1.65	KO up vs WT	
Flrt2	NM_201518	0.05	1.46	KO up vs WT	
Dbx1	NM_001005232	0.05	−2.35	KO down vs WT	Yes
Gpr56	NM_018882	0.05	1.56	KO up vs WT	
Efnb3	NM_007911	0.05	1.59	KO up vs WT	Yes
Nexn	NM_199465	0.05	1.72	KO up vs WT	
Ctgf	NM_010217	0.05	1.45	KO up vs WT	
Cp	NM_001042611	0.05	2.04	KO up vs WT	No
Ednrb	NM_007904	0.05	1.53	KO up vs WT	No

### Correlation between SOX3 Binding Sites and *Sox3* Targets

Differentially expressed genes in *Sox3* null NPs potentially represent genes that are direct SOX3 targets. To investigate this possibility, we examined the differentially expressed genes for SOX3 binding sites using a previously published SOX3 ChIP-seq data set generated using a comparable NP differentiation system [4]. A significant enrichment of SOX3 binding sites was present in genes with altered expression in *Sox3* null NP cells. Over 83% of the differentially expressed genes (Table 1) have at proximal (closest gene) SOX3 binding site (Fig. 2C). In contrast, SOX3 binding sites were detected in only 25% of 20 randomly selected genes that do not display altered expression in *Sox3* null NPs. The enrichment of SOX3 binding sites near genes with significantly altered expression genes suggests that many of these genes are direct targets of SOX3.

### In vivo Validation of SOX3 Targets

**Figure 3:**
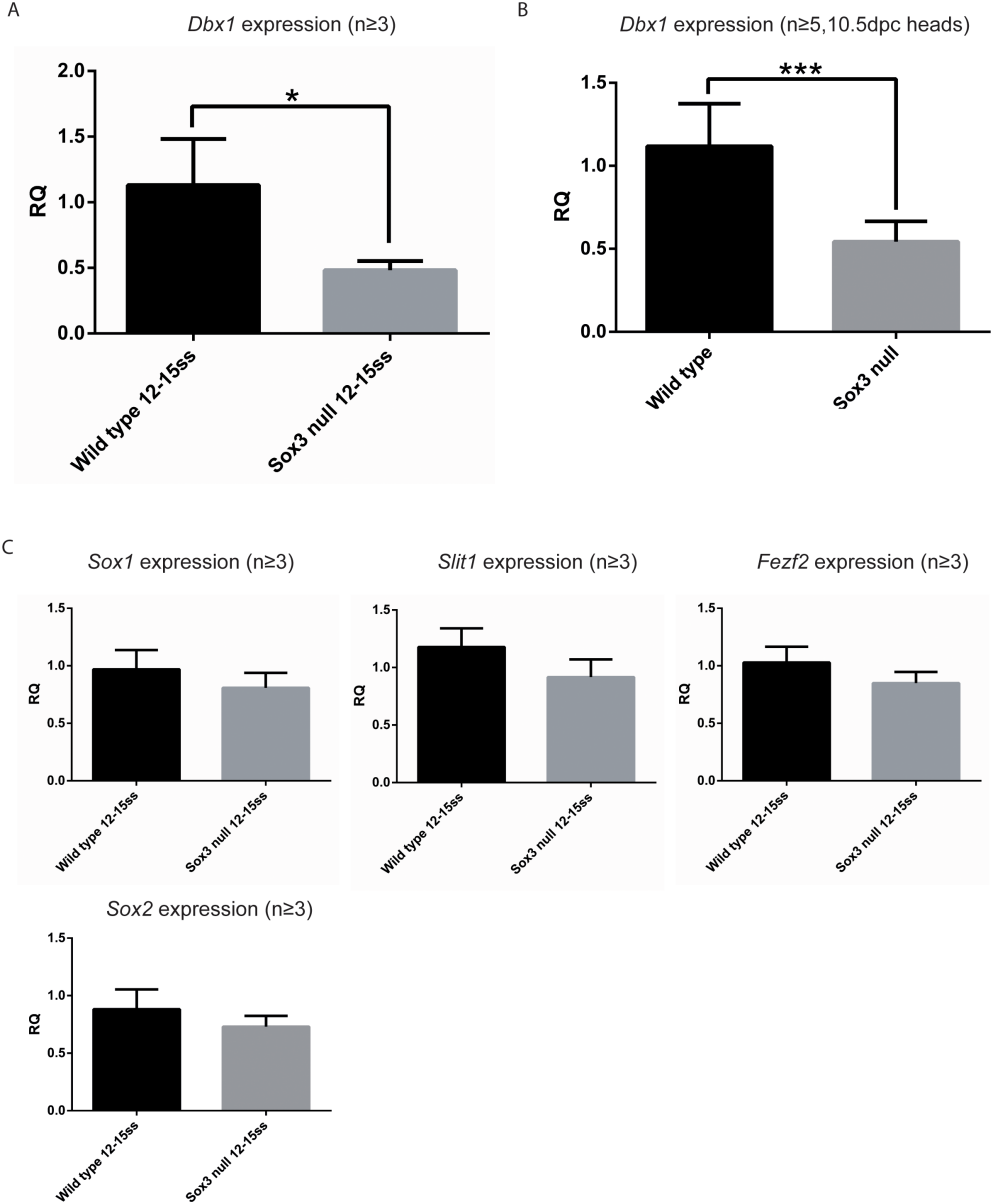
Validation of putative Sox3 targets *in vivo*. A: *Dbx1* gene expression within whole 9.5 dpc embryos. B: *Dbx1* gene expression within 10.5 dpc dissected heads. C: qRT-PCR analysis of mRNA levels of *Sox1*, *Sox2*, *Slit1* and *Fezf2* showing no significant difference. RQ: relative quantification normalised to β-actin.

Next, we investigated whether the differentially-expressed NP culture genes were affected by the loss of *Sox3* in vivo. This analysis was performed using 9.5 dpc embryos (somite stages 12–15) as embryos at this stage of development have an abundance of NP cells and a paucity of differentiating neurons. In addition, like Day 4 NP cell cultures, *Sox1* and *Sox2* expression in not significantly altered in *Sox3* null embryos at this stage (Fig. 3C). qRT-PCR analysis of *Slit1* and *Fezf2* showed no significant difference in expression between WT and *Sox3* null embryos at this developmental time point (Fig. 3C). In contrast, *Dbx1* expression was significantly lower in *Sox3* null embryos at 9.5 dpc (Fig. 3A), consistent with its downregulation in *Sox3* null NP cells. A significant reduction in *Dbx1* expression was also evident in 10.5 dpc embryonic heads (Fig. 3).

**Figure 4:**
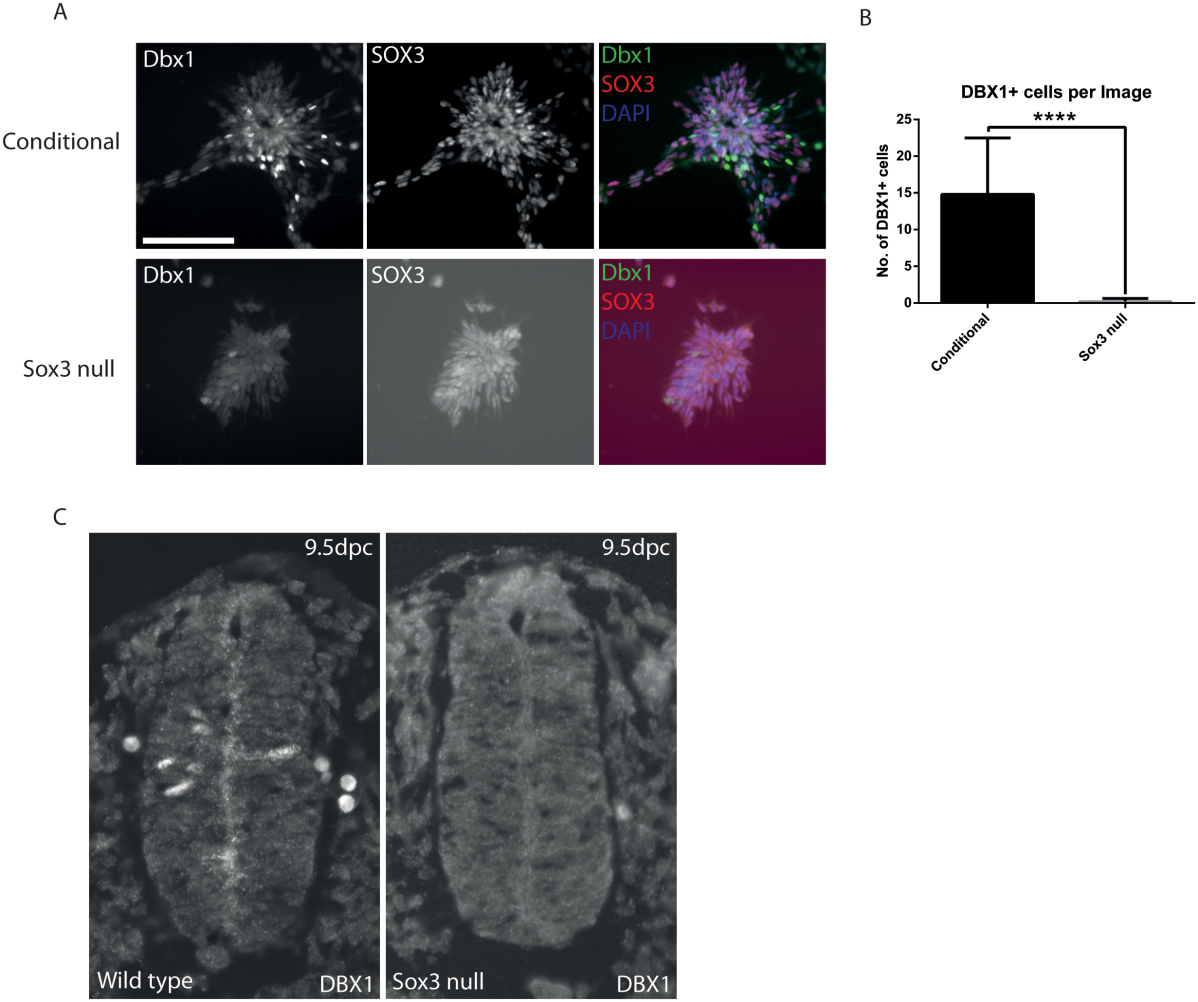
DBX1 levels within neural progenitor cells and the developing spinal cord. A: DBX1 expression at day 4 of N2B27 differentiation. B: Number of DBX1+ cells per field of view at day 4 of N2B27 differentiation. C: DBX1 expression within the developing 9.5 dpc spinal cord of wild type and *Sox3* null mice.

To determine the impact of *Sox3* loss-of-function on DBX1 expression at the cellular level, we compared the number of DBX1+ cells in Day 4 NP cultures using immunohistochemistry (Figure 4 A). DBX1+ cells were readily identified in control cultures and were located in a subpopulation of SOX3+ cells located at the periphery of cell clusters. In contrast, the number of DBX1+ cells was significantly and dramatically reduced in SOX3 KO cultures (Figure 4B). To determine whether there is a similar reduction in DBX1+ cells in *Sox3* mutant embryos, we compared DBX1 expression in 9.5 dpc transverse sections through the trunk region. Again, a clear reduction in the number of DBX1+ cells within the spinal cord of *Sox3* null mice was evident (Fig. 4C). Together these data suggest that *Dbx1* is regulated by SOX3 *in vivo* and that other SoxB1 factors cannot fully compensate for the loss of SOX3.

### SOX3 Binds to the *Dbx1* Locus In vivo

**Figure 5:**
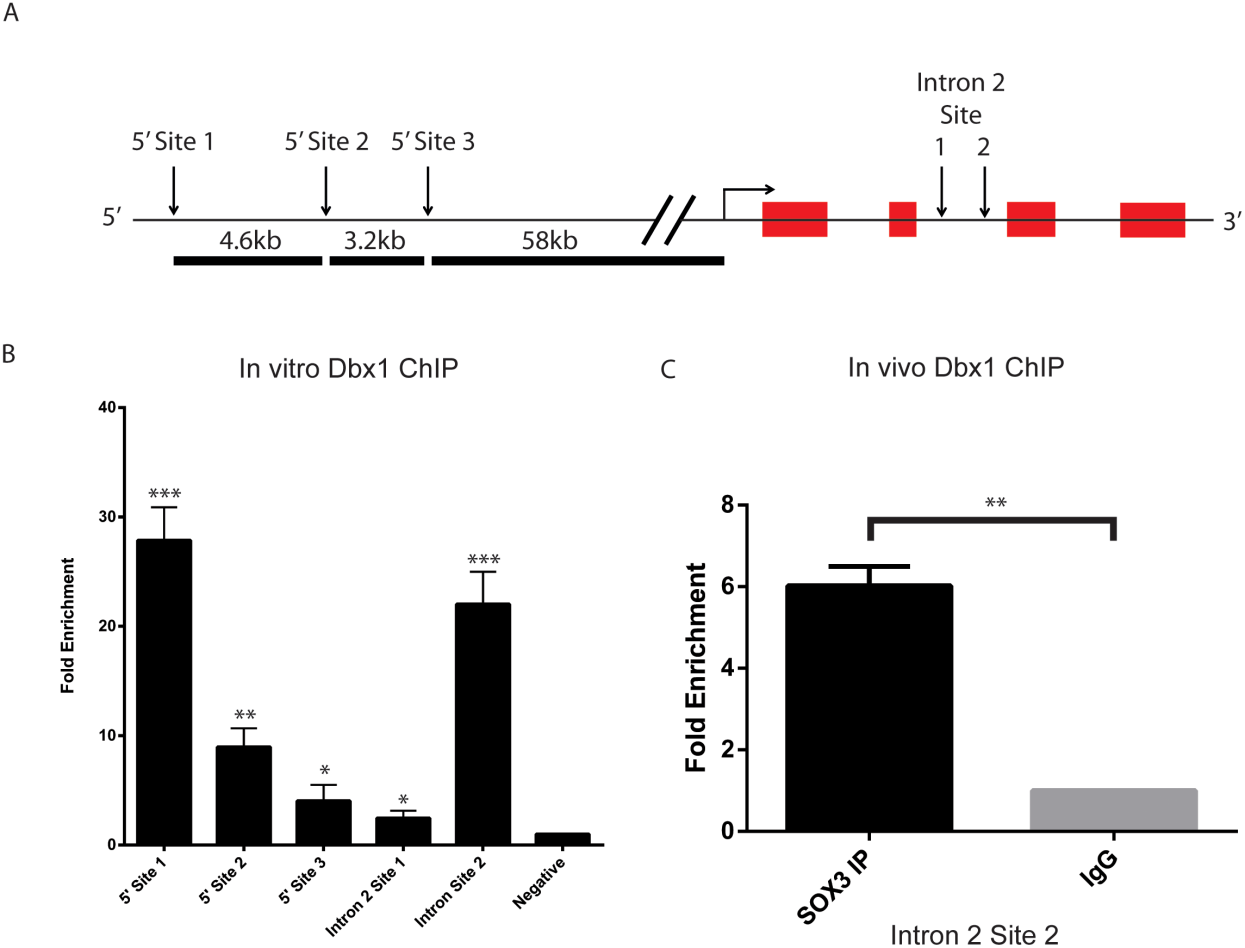
SOX3 binding sites surrounding *Dbx1*. A: Schematic diagram of previously published SOX3 binding sites (not to scale). B: *In vitro* ChIP validation of the five surrounding SOX3 binding sites (n = 3). C: *In vivo* ChIP validation of SOX3 binding to intronic site 2 in 10.5 dpc dissected heads (n = 3). All ChIP enrichment is relative to input and normalised to IgG IP.

To investigate whether *Dbx1* is directly targeted by SOX3 in Day 4 NP cells, we performed ChIP-PCR on five SOX3 binding sites located in or near the *Dbx1* locus that were previously identified by ChIP-seq analysis ([4]; Fig. 5A). Significant (p≤0.05) enrichment of all five sites was detected by SOX3 ChIP-PCR, when compared to the IgG isotype control (Fig. 5B). To determine if SOX3 bound these sites *in vivo*, we analysed SOX3 binding to one of the most enriched sites (Intronic site 2) using chromatin extracted from 10.5 dpc embryonic heads. Significant enrichment (p≤0.001) of this SOX3 binding this site was observed in comparison to the IgG negative control (Fig. 5C). Together, these data indicate that *Dbx1* is a direct target of SOX3 *in vivo*.

## Discussion

Despite the widespread expression of *Sox3* in progenitor cells throughout the neuroaxis, the CNS phenotypes of mice and humans with *Sox3* mutations are relatively mild. It is generally believed that other SOXB1 proteins (SOX1 and SOX2) can function redundantly with SOX3 to “rescue” the SOX3 deficiency. However, the lack of a complete phenotypic rescue suggests that, at least in some NP cells, the *level* of SOXB1 protein may be important for neurodevelopment. Alternatively, each of the SOXB1 factors could bind preferentially to a unique subset of SOXB1 targets. As a result, target gene expression resulting from the loss of a single SOXB1 gene would only be partially rescued by the other two SOXB1 factors. To explore this issue in more detail, we attempted to identify genes that were sensitive to the loss of SOX3 in NP cells. To do this we used a previously targeted *Sox3* null ES cell line in which the single SOX3 exon was replaced with GFP [17]. N2B27-induced generation of conditionally targeted and *Sox3* null NP cells resulted in rapid upregulation of *Sox3/GFP* at the mRNA and protein level. The NP markers *Sox1* and *Pax6* were also rapidly induced and showed comparable kinetics in the control and mutant cultures. In addition, expression of *Sox2* was comparable between the two populations. Notably, no overt defects in the morphology or global gene expression (see below) were identified in *Sox3* null NP cells. This is likely due to the expression of *Sox1* and *Sox2* in N2B27-induced NP cells and is consistent with the mild CNS phenotype of *Sox3* null embryos.

To maximise the likelihood of identifying genes that are directly regulated by SOX3, we performed gene expression profiling at Day 4, the earliest time point at which high levels of *Sox3* were expressed in the majority of NP cells. Of over 24,000 genes assayed by microarray, only 19 had significantly different expression levels >1.4-fold at Day 4. Expression differences (when tested by qRT-PCR) were validated on independent samples for a majority of the targets, thereby confirming the authenticity of the microarray data. However, not all differentially expressed genes validated on independent samples (*Tmem163, Ednrb, and Slc44a5,* data not shown). The relatively small number of differentially expressed genes is interesting to consider in the context of a recently-published ChIP-seq analysis of a similar NP cell type, which showed that SOX3 binds at thousands of genomic sites, many of which are likely to have regulatory functions [4]. Preliminary ChIP-seq analysis from our laboratory also indicates that SOX3 binds many of these sites in Day 4 N2B27-induced NP cells (D.M. and P.T. unpublished data). The most parsimonious explanation for the small number of differentially expressed genes is that SOX1 and/or SOX2 (both of which are expressed by Day 4 NP cells) can bind and functionally compensate for loss of SOX3. Consistent with this hypothesis, almost all SOX2 binding sites in NPs also bind SOX3 [4]. However, while SOXB1 redundancy provides an explanation for the unaltered expression of most genes associated with SOX3 binding sites, it also suggests that differentially expressed genes cannot be fully compensated by SOX1 and SOX2. Given the HMG domain of SOXB1 proteins is not identical, it is possible that they have subtly different preferences for sequence-specific binding in vivo, as has been suggested by in vitro DNA binding experiments [18]. In addition, amino acid differences within the HMG box (or outside of it) may influence interactions with DNA binding partners such as the POU-class transcription factors [19], [20]. Regardless of the mechanism, it is important to note that the differentially expressed genes are highly enriched for SOX3 binding sites (over randomly selected genes without significantly different expression levels), supporting the idea that they represent direct SOX3 targets. As a number of these putative direct target genes are upregulated, it appears that that SOX3 could function as a repressor in NP cells [21] in addition to its more established role as an activator [2].

Interestingly, many of the genes identified by the microarray analysis have established roles in neurodevelopment. For example, *Cspg5* has been associated with intellectual disability disorders [22], and ectopic *Slit1* expression has been published in mice with abnormal corpus callosum development [23]. Intellectual disability is present in a subset of individuals with *SOX3* mutations [7], while *Sox3* null mice have abnormal corpus callosum development [5], thereby providing a link between these putative targets and processes with a functional requirement for SOX3.

Another putative SOX3 target was the downregulated transcription factor gene *Fezf2. Fezf2* has been shown to be important for the early development of the posterior hypothalamus/thalamus in zebrafish and mouse [24], [25]. *Fezf2* expression has been previously published to be downstream of the SOXC group genes *Sox4* and *Sox11*, double deletion of which leads to a loss of *Fezf2* expression. Regulation of *Fezf2* by SOX4/11 gene is mediated by a *cis* acting enhancer binding site which, interestingly, was also identified as a SOX3 binding site in NP cells by ChIP-seq and independently within our lab (data not shown; [4], [26]). These data suggest that, in addition to acting as a pioneer factor [4], *Sox3* may directly regulate *Fezf2* expression in NP cells.

### *Dbx1* is a Direct SOX3 Target In vivo

To investigate whether NP targets were dysregulated in vivo, we compared their expression by qRT-PCR using whole 9.5 dpc embryos. Of the seven putative targets analysed (Fig. 3C, data not shown), only one (*Dbx1*) had significantly different expression that matched the genome-wide expression profiling analysis. The lack of *in vivo* validation for other targets may reflect a phenotypic or stage-specific difference in the NP cells induced by N2B27 differentiation compared with their 9.5 dpc embryonic counterparts. Consistent with this idea, expression analysis of *Sox3* targets in Day 6 NP cells revealed that many genes no longer had significantly different expression levels or had greatly reduced expression fold changes (Fig. S1). *Dbx1* expression, however, was reduced in whole 9.5 dpc embryos lacking *Sox3*. In addition, DBX1+ cells were not detected in the spinal cord of *Sox3* null embryos at 9.5 dpc and were massively reduced in Day 4 null NP cells. Further, SOX3 bound to evolutionarily conserved elements at the *Dbx1* locus in cultured NP cell and embryos. Together these data indicate that *Dbx1* is directly activated by SOX3 in NP cells.

*Dbx1* encodes a homeoprotein that is transiently expressed in Vo interneuron precursors and required for Vo interneuron differentiation [27]. The lack of DBX1 expression in the spinal cord of *Sox3* null embryos is the first clear evidence that there is a phenotype within the developing spinal cord of *Sox3* null mice and raises the possibility that generation of Vo interneurons is abnormal in *Sox3* mutants [5]. The significantly reduced DBX1 expression in *Sox3* null spinal cord NPs also provides some molecular insights into SOXB1 functional redundancy. Given that the spinal cord NPs have virtually identical SOXB1 expression [2], [14], it appears that SOX1 and SOX2 are unable to substitute for SOX3 in the context of *Dbx1* regulation. As discussed above, this might reflect preferential binding of SOX3 at the *Dbx1* locus. Alternatively, *Dbx1* expression could be sensitive to the overall dosage of SOXB1 protein. At least two experimental approaches could be used to test these possibilities. Firstly, it would be interesting to examine *Dbx1* expression in *Sox1* and *Sox2* single gene KO NP cells ie. under conditions where the overall SOXB1 dosage is lower but SOX3 is present. Secondly, a targeted gene replacement strategy (eg. replacing *Sox3* with *Sox2*) could be used to generate NP cells that have an equivalent SOXB1 dosage but which lack SOX3. In addition to the spinal cord, we identified a reduction in *Dbx1* expression in the head of 10.5 dpc *Sox3* null embryos. However, we were unable to determine whether this reflected a lack of *Dbx1* expression in a specific region or general reduction in the *Dbx1* expression level (data not shown).

A recent report by Oosterveen et al also indicated that *Dbx1* is a direct target of Sox3 [28]. However, the SOX3 binding sites reported by Oosterveen et al are contained within a putative cis-regulatory module (CRM) that does not overlap with the SOX3 binding sites identified in this study. The Oosterveen et al data raises the possibility that (the lack of) SOX3 binding at the CRM sites may contribute to the reduction of *Dbx1* expression in *Sox3* null NPs. The data also suggest that the regulation of *Dbx1* by SOX3 is complex and subtle differences between early or later expression or spinal cord/brain expression might be regulated any number of these sites. Further studies are needed to address this issue. Given the increasing ease with which the genome can be manipulated [29], it would be interesting to perform systematic mutagenesis of all known SOXB1 sites at the *Dbx1* locus and assess their impact on *Dbx1* expression *in vitro* and *in vivo*.

## Supporting Information

Figure S1 **Expression of putative Sox3 targets at day 6 of neural progenitor differentiation.** A: Putative targets *Dbx1* and *Slit1* relative gene expression levels by qRT-PCR at day 6 of N2B27 differentiation, (n = 3 normalised to day 4). B: Putative targets *Cp, Fezf2* and *Efnb3* showing reduced and/or insignificant fold change at day 6 of N2B27 differentiation (n = 3, normalised to day 4). RQ: relative quantification normalised to β-actin.
(TIF)
