# Supplementary figures and images for: Dbx1 Is a Direct Target of SOX3 in the Spinal Cord
Source: PLoS One. 2014 Apr 21;9(4):e95356. doi: 10.1371/journal.pone.0095356 (PMC3994032; doi:10.1371/journal.pone.0095356)

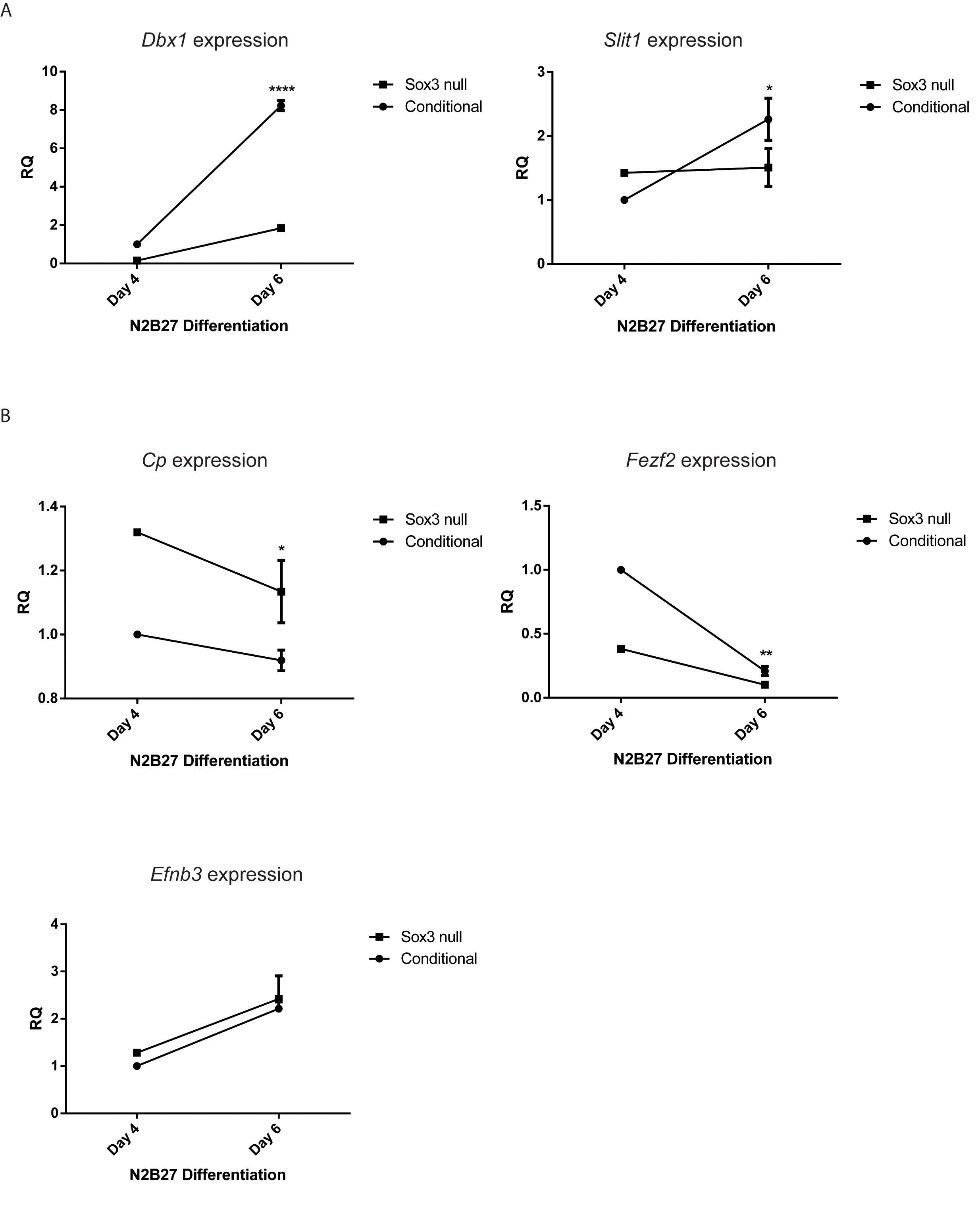

Supplement: Figure S1 — Expression of putative Sox3 targets at day 6 of neural progenitor differentiation. A: Putative targets Dbx1 and Slit1 relative gene expression levels by qRT-PCR at day 6 of N2B27 differentiation, (n = 3 normalised to day 4). B: Putative targets Cp, Fezf2 and Efnb3 showing reduced and/or insignificant fold change at day 6 of N2B27 differentiation (n = 3, normalised to day 4). RQ: relative quantification normalised to β-actin. (TIF) [file pone.0095356.s001.tif]
